# Proceedings of the Medical Convention of the State of Tennessee Held in Nashville, Oct. 1847

**Published:** 1848-02

**Authors:** 


*Proceedings of the Medical Convention of the State of Tennessee held in Nashville, Oct.* 1847.

We have been favored with a pamphlet containing the proceedings of a Medical Convention held at Nashville, Tenn., in which resolutions were passed approving of the report of the Committee on Education to the late National Convention, and pledging all honorable support to Colleges which are governed by the resolutions attached to the report referred to.

The code of medical ethics recommended by the National Convention was adopted. A committee was appointed to procure the passage of laws respecting the registration of births, marriages and deaths. Several resolutions were also adopted with respect to the importance of preliminary education, and of primary medical schools in which the pupil may be prepared to enter Medical Colleges.

